# Aftershocks Following the 9 April 2013 Bushehr Earthquake, Iran

**DOI:** 10.1371/currents.dis.76750ede500e61b81d7f2ba9edfb2373

**Published:** 2013-08-28

**Authors:** Ali Ardalan, Alireza Hajiuni, Mehdi Zare

**Affiliations:** Department of Disaster & Emergency Health, Iran's National Institute of Health Research; Department of Disaster Public Health, School of Public Health, Tehran University of Medical Sciences, Tehran, Iran; Harvard Humanitarian Initiative, Harvard University; Department of Disaster Public Health, School of Public Health, Tehran University of Medical Sciences, Tehran, Iran; Department of Disaster & Emergency Health, National Institute of Health Research, Tehran University of Medical Sciences, Tehran, Iran; Engineering Seismology / National Center for Earthquake Prediction, International Institute of Earthquake Engineering and Seismology (IIEES), Tehran, Iran

## Abstract

On 9 April 2013 at 11:52 UTC (16:22 local time), a Mw 6.2 earthquake occurred at the depth of 20 Km in Dashti district in south-west Iran’s Bushehr province. The macroseismic epicenter was located nearby the city of Shonbeh. During one month after the earthquake, a total of 282 aftershocks hit the epicentral region, mostly at the east and north sides. They ranged from 2.5 to 5.7 on the Richter scale. Seventy aftershocks (24.9%) were M4.0-4.9 and eight (2.8%) were M5.0-5.7. Aftershocks are potentially able to do additional damage. In Bushehr earthquake, a M5.4 aftershock on 10 April in Chahgah village caused at least four injuries and destruction of several buildings that had been already damaged by the main shock. Knowledge about the aftershock induced damages provides opportunities for timely risk communication with the affected people and for long term community education. This will hopefully increase the community awareness and minimize the risk of further loss of lives.

## Brief Incident Report

On 9 April 2013 at 11:52 UTC (16:22 local time), a Mw 6.2 earthquake occurred at the depth of 20 Km in Dashti district in south-west Iran’s Bushehr province 1. The macroseismic epicenter was located near the city of Shonbeh. The earthquake caused 37 deaths and 850 injuries. Over 1,000 houses in 92 villages were damaged. The earthquake resulted from thrust faulting on a NW-SE trending fault plane, consistent with continuing shortening of the Arabian Plate 2.

According to the International Institute of Earthquake Engineering and Seismology based in Tehran, during one month following the earthquake, a total of 282 aftershocks hit the epicentral region 1. The aftershocks occurred mostly at the east and north of the epicenter 3 and ranged from 2.5 to 5.7 on the Richter scale (RS). The distribution of aftershocks is representative for principal stress direction (NE-SW).

Distribution of aftershocks’ magnitude on the RS was as follow: 33 M<3.0 (11.7%), 171 M3.0-3.9 (60.6%), 70 M4.0-4.9 (24.9%) and 8 M5.0-5.7 (2.8%). Among the 78 aftershocks with M ≥4.0, four aftershocks (5.2%) occurred at the depth of ≤10 Km, 63 (80.8%) at the depth of 11-39 Km, seven (8.9%) at the depth of 40-50 Km, three (3.9%) at the depth of 50-100 Km and one aftershock (1.2%) at the depth of 102 Km 1. Figure 1 illustrates the aftershocks geographical distribution and figure 2 shows a decreasing trend of the aftershocks in terms of number and magnitude over 30 days after the main shock.

**Geographical distribution of aftershocks following 9 April 2013 Bushehr earthquake:**
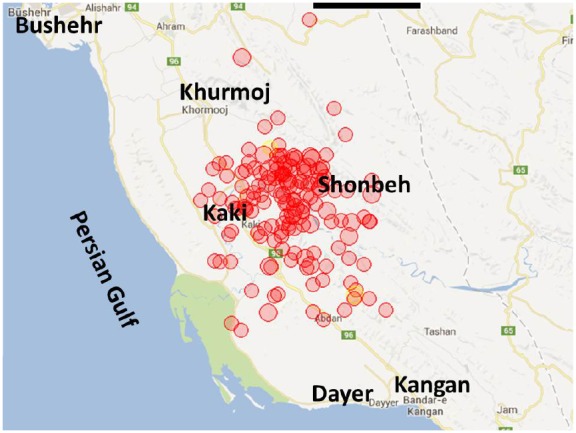
(Source: International Institute of Earthquake Engineering and Seismology)

**Number of aftershocks within 30 days of 9 April 2013 Bushehr earthquake by magnitude on the Richter scale:**
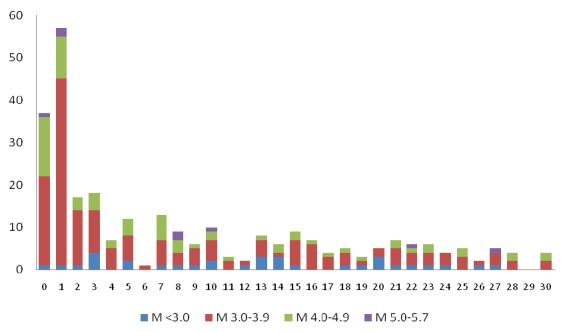
(Source: International Institute of Earthquake Engineering and Seismology)

By definition, aftershocks are earthquakes that follow the largest shock of an earthquake sequence 4. They are smaller than the main shock in size and occur as the touching edges of two plates need to adjust to new positions. This adjustment may takes weeks, months, or years. The location of the aftershocks, however, is not predictable. But, as a general rule, they occur within a characteristic distance from the main shock that is usually taken to be one or two times the length of the rupture associated with the main shock 5.

The aftershocks are potentially able to do additional damage 6. While, any felt aftershock may cause additional damage or create new falling hazards, the risk is higher with those of magnitude 5.0 and larger. The damage, of course, also depends on the site conditions, building type, and distance from the aftershock. This risk is highest for previously damaged buildings 5. Among the aftershocks’ impacts, we remember the collapse of a hotel that caused 44 deaths following the Van earthquake of 23 October 2011 7. A magnitude 5.8 earthquake on 29 May 2012 in Italy is another example. It hit the epicenter region nine days after the main shock and caused an additional 20 deaths and widespread damage, particularly to buildings already weakened by the 20 May earthquake 8.

In Bushehr earthquake, a M5.4 aftershock on 10 April in Chahgah village caused at least four injuries and destruction of several buildings that had been already damaged by the main shock on 9 April, according to the Director of Iran’s National Disaster Management Organization 9. We could not find any other official report or statement regarding the other impacts of the aftershocks in this earthquake.

Lack of a consolidated reporting system on consequences of aftershocks is a challenge as we have also observed in other major earthquakes in Iran, like Bam (2003), Zarand (2005) and Lorestan (2006) earthquakes. This limited our knowledge about the aftershocks induced damages which is necessary for timely risk communication with the affected people and for a long term community education. Because people entering damaged buildings are at risk should an aftershock occur, we believe that this information must be developed and shared with Iranian community to increase their awareness and minimize the risk of further loss of lives.

## Competing Interests

The authors have declared that no competing interests exist.

## Corresponding Author

Ali Ardalan MD, PhD. Tehran University of Medical Sciences. Harvard Humanitarian Initiative. Email: ardalan@hsph.harvard.edu
